# Interdisciplinary Management of Advanced Periodontitis With Severe Anterior Alveolar Ridge Deficiency Using Periodontally Accelerated Osteogenic Orthodontics and Titanium Mesh-Guided Bone Regeneration: A Case Report

**DOI:** 10.7759/cureus.117136

**Published:** 2026-09-29

**Authors:** Evelin M Mejia Ocampo, Elizandra Valladares, Marcelo Romero, Ana M Valle Rivas, Maryory Perez Quevedo

**Affiliations:** 1 Dentistry, Independent Research, Indianapolis, USA; 2 Periodontics, Advanced Dental Group, San Pedro Sula, HND; 3 Dentistry, Calderon Meron Odontologia Especializada, Mérida, MEX; 4 Dentistry, Armando Avila Dental Office, Indianapolis, USA; 5 Dentistry, Private Practice, Dallas, USA

**Keywords:** advanced periodontitis, alveolar ridge deficiency, case report, corticotomy, guided bone regeneration, interdisciplinary treatment, orthodontics, paoo, periodontally accelerated osteogenic orthodontics, titanium mesh

## Abstract

Advanced periodontitis with severe anterior alveolar ridge deficiency can complicate orthodontic tooth movement and subsequent rehabilitation. This report describes the interdisciplinary management of a 46-year-old woman diagnosed with generalized Stage III Grade B periodontitis, severe anterior maxillary alveolar ridge deficiency, pathological migration of the maxillary anterior teeth, and multiple missing teeth.

Initial nonsurgical periodontal therapy was performed before surgical intervention. After clinical periodontal stability was achieved, the patient underwent periodontally accelerated osteogenic orthodontics (PAOO) consisting of selective alveolar corticotomies combined with guided bone regeneration (GBR) using a particulate bone graft and titanium mesh for space maintenance. Controlled orthodontic treatment was subsequently initiated, and supportive periodontal therapy was provided at three-month intervals.

Serial clinical photographs and conventional radiographs documented maintenance of the augmented anterior ridge contour, progressive orthodontic alignment, and periodontal stability during the available follow-up. Strategically maintainable teeth were preserved, and implant-supported rehabilitation remained planned as a future restorative phase. Because standardized three-dimensional measurements were unavailable, the magnitude and composition of regenerated bone could not be quantified.

This case documents a carefully sequenced approach integrating periodontal stabilization, regenerative surgery, orthodontic treatment, and supportive periodontal maintenance. The observations are limited to a single patient and should not be interpreted as proof of predictable treatment effectiveness.

## Introduction

Advanced periodontitis is a chronic inflammatory disease characterized by progressive destruction of the tooth-supporting tissues, resulting in clinical attachment loss, alveolar bone resorption, pathological tooth migration, occlusal instability, functional impairment, and ultimately tooth loss if left untreated [1-3]. Patients presenting with advanced periodontal destruction frequently require an interdisciplinary approach involving periodontics, orthodontics, oral and maxillofacial surgery, implant dentistry, and restorative rehabilitation. Contemporary periodontal care emphasizes control of inflammation, preservation of strategically maintainable teeth, correction of pathological tooth migration, and long-term supportive periodontal therapy [4].

Periodontally accelerated osteogenic orthodontics (PAOO) combines selective alveolar corticotomy with particulate bone grafting. The surgically induced regional acceleratory phenomenon (RAP) produces a temporary increase in bone turnover that may facilitate controlled orthodontic tooth movement, whereas the graft is intended to support the alveolar housing [5-8]. In severe horizontal or vertical ridge deficiencies, guided bone regeneration (GBR) requires exclusion of rapidly proliferating soft tissue, stabilization of the blood clot and graft, maintenance of regenerative space, and adequate vascular supply [9-13]. A titanium mesh can provide rigid space maintenance and preserve the intended contour and stability of a particulate graft, although the outcome remains dependent on soft-tissue closure, wound stability, host biology, and plaque control [14].

In adults with periodontal disease, combined periodontal and orthodontic treatment requires coordinated risk assessment and continued supportive periodontal therapy because maintenance care is essential for preserving periodontal stability [15-17].

Alveolar bone healing is governed by more than scaffold mechanics. The early inflammatory response, macrophage and other immune-cell behavior, angiogenesis, recruitment and differentiation of osteogenic cells, and subsequent remodeling collectively influence the host response to a grafted site. Scaffold architecture, porosity, surface characteristics, biocompatibility, mechanical stability, and tissue integration can affect cell attachment, vascular ingrowth, and bone formation [18]. In a periodontally compromised environment, persistent dysbiosis and inflammation may sustain osteoclastogenic signaling and impair osteogenic repair. Current biomaterial strategies therefore increasingly seek to regulate the local inflammatory and osteoimmune microenvironment in addition to providing structural support [19]. In the present case, the particulate graft and titanium mesh were used as conventional osteoconductive and space-maintaining components; no intrinsic immunomodulatory effect of either material was measured.

The specific clinical interest of this report is the sequential integration of periodontal stabilization, PAOO, titanium mesh-supported GBR, orthodontic treatment, and supportive periodontal therapy in a patient with generalized Stage III Grade B periodontitis and severe anterior maxillary ridge deficiency. The rationale was to control inflammation before regenerative and orthodontic intervention, preserve strategically maintainable teeth, maintain an augmented ridge contour during healing, and create more favorable anatomical conditions for controlled tooth movement and future restorative planning. This report documents the clinical sequence and available follow-up; it does not quantify regenerated bone or establish the efficacy or predictability of this approach.

## Case presentation

Patient information 

A 46-year-old woman presented to the Periodontology Department in March 2017, seeking treatment for progressive mobility of the maxillary anterior teeth, increasing interdental spacing, and deterioration of smile esthetics. Her primary concern was preservation of her natural dentition, and she stated, “I want to keep my teeth.”

Medical and dental history 

The patient’s medical history was noncontributory, with no systemic diseases known to interfere with periodontal healing or bone metabolism. She reported a documented allergy to clindamycin, denied tobacco use, and had no history of endocrine, cardiovascular, neurological, gastrointestinal, or infectious diseases that could compromise treatment outcomes. 

The patient had previously received routine dental care, including prophylaxis, restorative treatment, tooth extractions, periodontal therapy, and supportive periodontal maintenance. Two weeks before presentation, she attended an orthodontic consultation; however, comprehensive periodontal evaluation was recommended before orthodontic treatment because of the severity of her periodontal condition. 

Clinical examination 

Extraoral examination revealed facial symmetry, with no cervical lymphadenopathy or signs of temporomandibular joint dysfunction. Intraoral examination demonstrated generalized inflammatory periodontal changes characterized by flattened interdental papillae, loss of normal gingival scalloping, generalized gingival recession with root-surface exposure, and reduced gingival volume. A thick periodontal phenotype was observed, and the baseline clinical presentation is shown in Figure 1.

**Figure 1:**
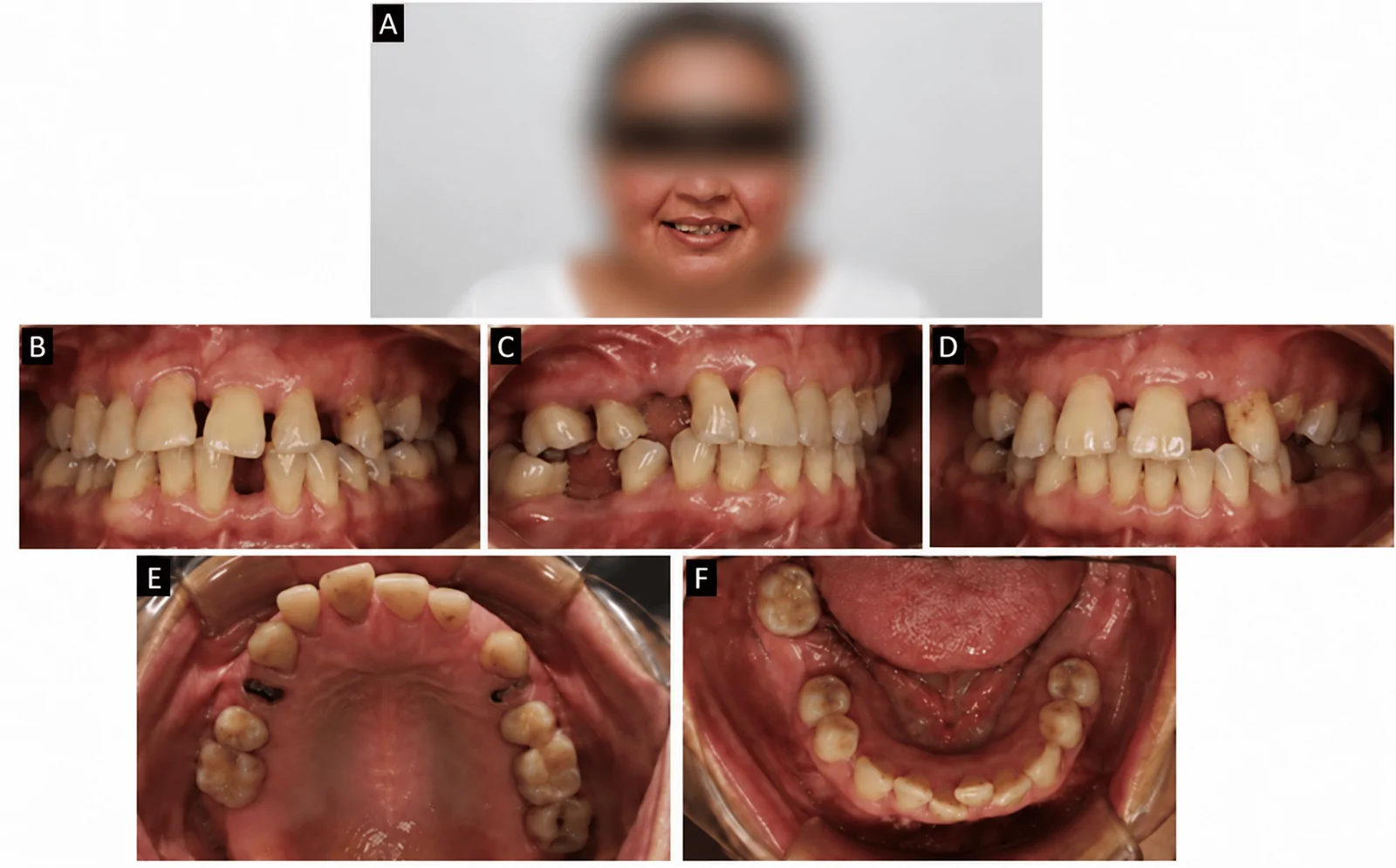
Baseline clinical presentation (A) Anonymized frontal facial view; (B) Frontal intraoral view in maximum intercuspation; (C) Right lateral view; (D) Left lateral view; (E) Maxillary occlusal view; (F) Mandibular occlusal view. The patient provided written and signed consent, allowing publication of this identifiable facial image in an open-access journal.

Additional clinical findings included generalized plaque accumulation, pathological migration and spacing of the maxillary anterior teeth, occlusal instability, and multiple missing teeth. The baseline O'Leary Plaque Control Record was 70%, indicating inadequate oral hygiene at the initial examination. The baseline periodontal chart documented generalized probing depths, bleeding on probing, plaque accumulation, and gingival-margin measurements; however, tooth-specific clinical attachment levels and mobility grades were not consistently available in the records. The baseline periodontal chart and diagnostic summary are presented in Figure 2.

**Figure 2:**
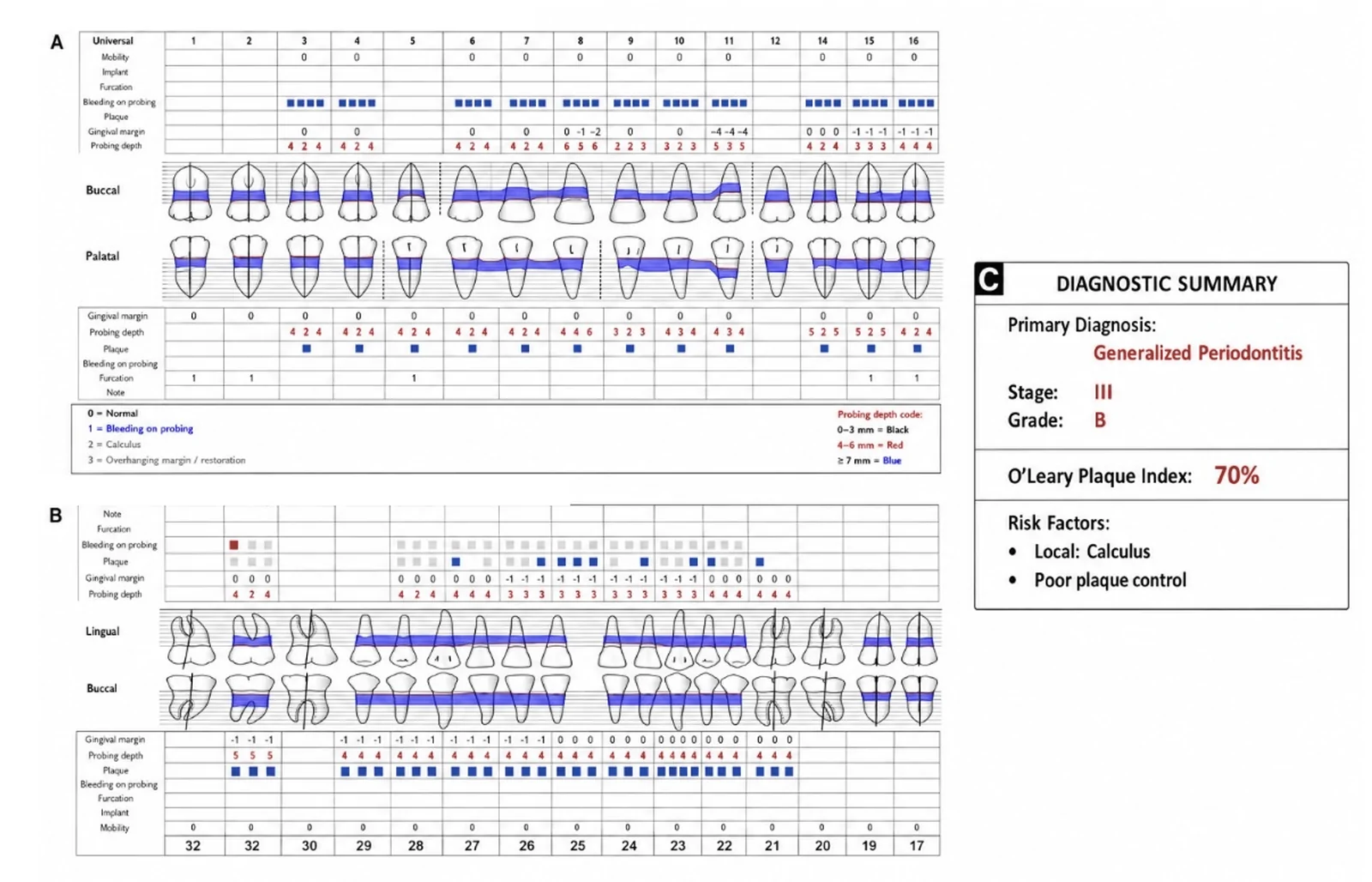
Baseline periodontal evaluation demonstrating generalized periodontal involvement and inadequate plaque control (A) Maxillary periodontal chart showing generalized probing depths, bleeding on probing, plaque accumulation, and gingival margin measurements; (B) Mandibular periodontal chart demonstrating generalized probing depths, bleeding on probing, plaque accumulation, and gingival margin measurements; (C) Diagnostic summary showing generalized Stage III Grade B periodontitis, an O'Leary Plaque Control Record of 70%, local calculus, and poor plaque control.

Periodontal and radiographic examination 

Comprehensive periodontal examination revealed generalized clinical attachment loss, with probing depths ranging from 3 to 5 mm and multiple sites exhibiting probing depths greater than 5 mm. The anterior maxillary region exhibited severe horizontal and vertical alveolar ridge deficiency, pathological tooth migration, loss of interdental papillary support, and inadequate bone volume surrounding the remaining teeth. These findings represented the principal biological limitations for orthodontic tooth movement and future implant-supported rehabilitation. 

Baseline panoramic and full-mouth periapical radiographs demonstrated generalized horizontal alveolar bone loss with localized vertical osseous defects, particularly affecting the anterior maxilla. Multiple teeth exhibited advanced periodontal support loss, while the anterior maxillary ridge presented marked reduction in bone volume. The baseline radiographic findings are shown in Figure 3.

**Figure 3:**
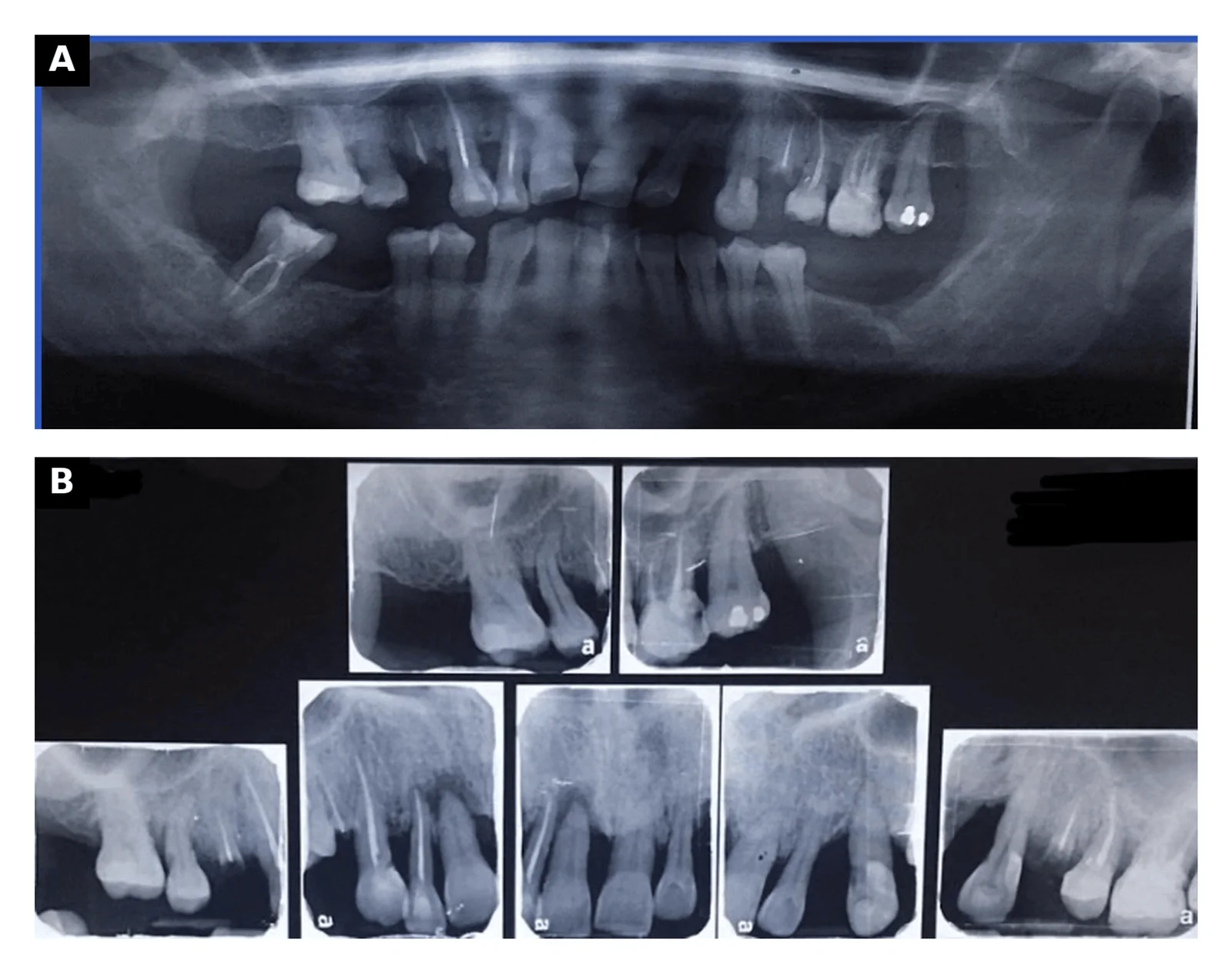
Initial radiographic assessment demonstrating generalized horizontal alveolar bone loss, localized vertical osseous defects, and severe anterior maxillary alveolar ridge deficiency associated with advanced periodontal destruction (A) Baseline panoramic radiograph; (B) Representative baseline periapical radiographs of the anterior maxillary region.

Diagnostic assessment 

Based on the comprehensive clinical, periodontal, and radiographic findings, the patient was retrospectively diagnosed according to the 2017 World Workshop classification system with generalized Stage III Grade B periodontitis. The Stage III classification was supported by generalized clinical attachment loss, radiographic bone loss extending to the middle third of the roots and beyond at several sites, localized vertical osseous defects, pathological tooth migration, occlusal instability, and documented tooth loss attributed to periodontal destruction. The condition was classified as generalized because more than 30% of the remaining teeth were affected. 

Grade B was assigned retrospectively from the available radiographic bone-loss pattern relative to the patient’s age and the absence of smoking and uncontrolled diabetes as grade modifiers. Serial measurements adequate to calculate a direct five-year progression rate were not available [2,3]. The complete assessment comprised generalized Stage III Grade B periodontitis, severe horizontal and vertical anterior maxillary alveolar ridge deficiency, pathological migration of the maxillary anterior dentition, occlusal instability secondary to periodontal attachment loss, and multiple missing teeth requiring future implant-supported rehabilitation. 

These findings indicated that conventional orthodontic treatment alone would not be biologically appropriate. Consequently, an interdisciplinary treatment strategy emphasizing periodontal stabilization, alveolar ridge reconstruction, controlled orthodontic tooth movement, and continuing supportive periodontal therapy was selected. 

Clinical timeline 

The baseline periodontal and radiographic assessment was performed in March 2017. The initial phase included oral-hygiene instruction, full-mouth scaling and root planing, management of local factors, occlusal adjustment when indicated, and extraction of hopeless teeth. After periodontal reevaluation, selective corticotomies and GBR with a particulate graft and titanium mesh were performed. Postoperative assessments occurred at one day, eight days, and three months; the mesh was removed nine months after surgery, and orthodontic follow-up included a documented four-month evaluation with supportive periodontal therapy at three-month intervals. 

Interdisciplinary treatment planning 

The primary objective was to preserve the patient’s natural dentition while restoring periodontal health, correcting pathological tooth migration, reconstructing the deficient anterior alveolar ridge, and establishing favorable conditions for future restorative rehabilitation. Because active periodontal inflammation and severe alveolar bone loss represented the principal biological limitations, treatment was organized into sequential phases so that each intervention could be performed under appropriate clinical conditions. 

Phase I consisted of oral-hygiene instruction, patient motivation, full-mouth scaling and root planing, extraction of hopeless teeth when indicated, occlusal adjustment as required, and periodontal reevaluation after resolution of inflammation. Phase II consisted of PAOO and GBR, including elevation of a full-thickness mucoperiosteal flap, selective alveolar corticotomies, placement of a particulate bone graft, adaptation of titanium mesh for space maintenance, and tension-free primary wound closure. 

Phase III involved controlled orthodontic tooth movement after confirmation of periodontal stability and satisfactory healing, followed by progressive correction of pathological tooth migration and optimization of tooth position. Phase IV consisted of supportive periodontal therapy at three-month intervals, continuous monitoring of periodontal stability and the regenerated ridge, and planning for future implant-supported rehabilitation after completion of periodontal and orthodontic therapy. 

Therapeutic intervention 

Treatment began with comprehensive nonsurgical periodontal therapy aimed at eliminating active inflammation and establishing a stable periodontal environment before surgical and orthodontic intervention. Initial therapy included individualized oral-hygiene instruction, patient motivation, full-mouth scaling and root planing, management of local contributing factors, occlusal adjustment when indicated, and extraction of teeth considered periodontally hopeless. 

At periodontal reevaluation, the available records documented reduced clinical inflammation and improved plaque control. A complete numerical post-treatment periodontal chart, including tooth-specific probing depths, clinical attachment levels, bleeding on probing, mobility grades, and a repeat full-mouth plaque score, was unavailable. Periodontal stability was therefore determined clinically before regenerative surgery, and the absence of progressive attachment loss could not be quantified. Surgical exposure and full-thickness flap elevation are shown in Figure 4.

**Figure 4:**
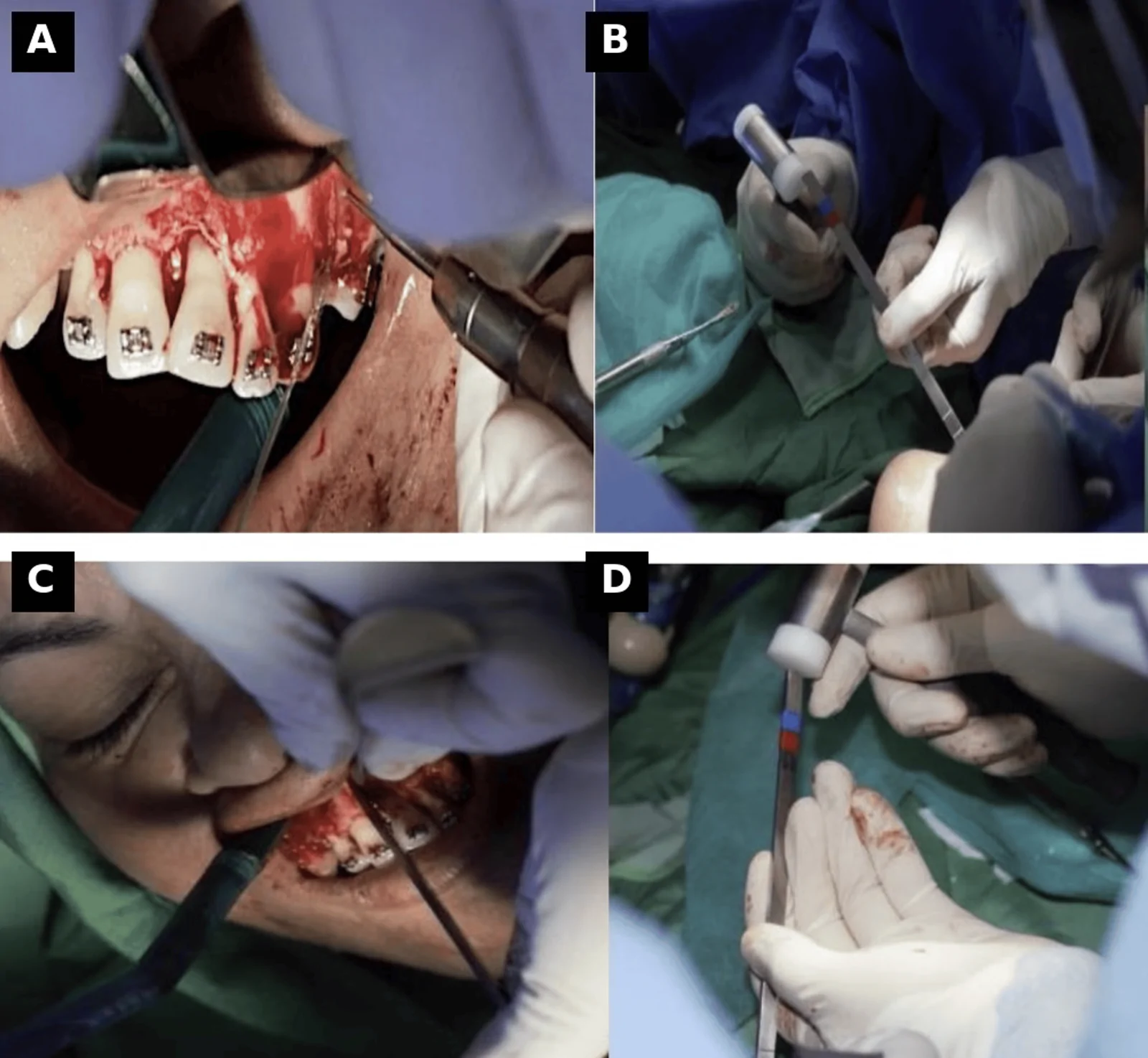
Surgical exposure and full-thickness flap elevation (A) Initial surgical exposure of the anterior maxillary region; (B, C) Elevation of the full-thickness mucoperiosteal flap; (D) Exposure of the buccal cortical plate following flap reflection.

Because of the severe horizontal and vertical deficiency of the anterior maxillary ridge and the need to facilitate controlled orthodontic tooth movement within a compromised periodontal environment, PAOO combined with GBR was selected [6-8,10]. Under local anesthesia, a full-thickness mucoperiosteal flap was elevated in the anterior maxilla through a scalloped crestal incision with vertical releasing incisions distal to the canines. This design provided adequate access to the facial alveolar bone while preserving the interdental papillae. 

Selective alveolar corticotomies were performed using vertical interradicular cuts connected by horizontal corticotomies extending beyond the root apices. Continuous sterile saline irrigation was maintained throughout the procedure to prevent overheating and thermal injury to the alveolar bone. The completed corticotomy pattern is shown in Figure 5.

**Figure 5:**
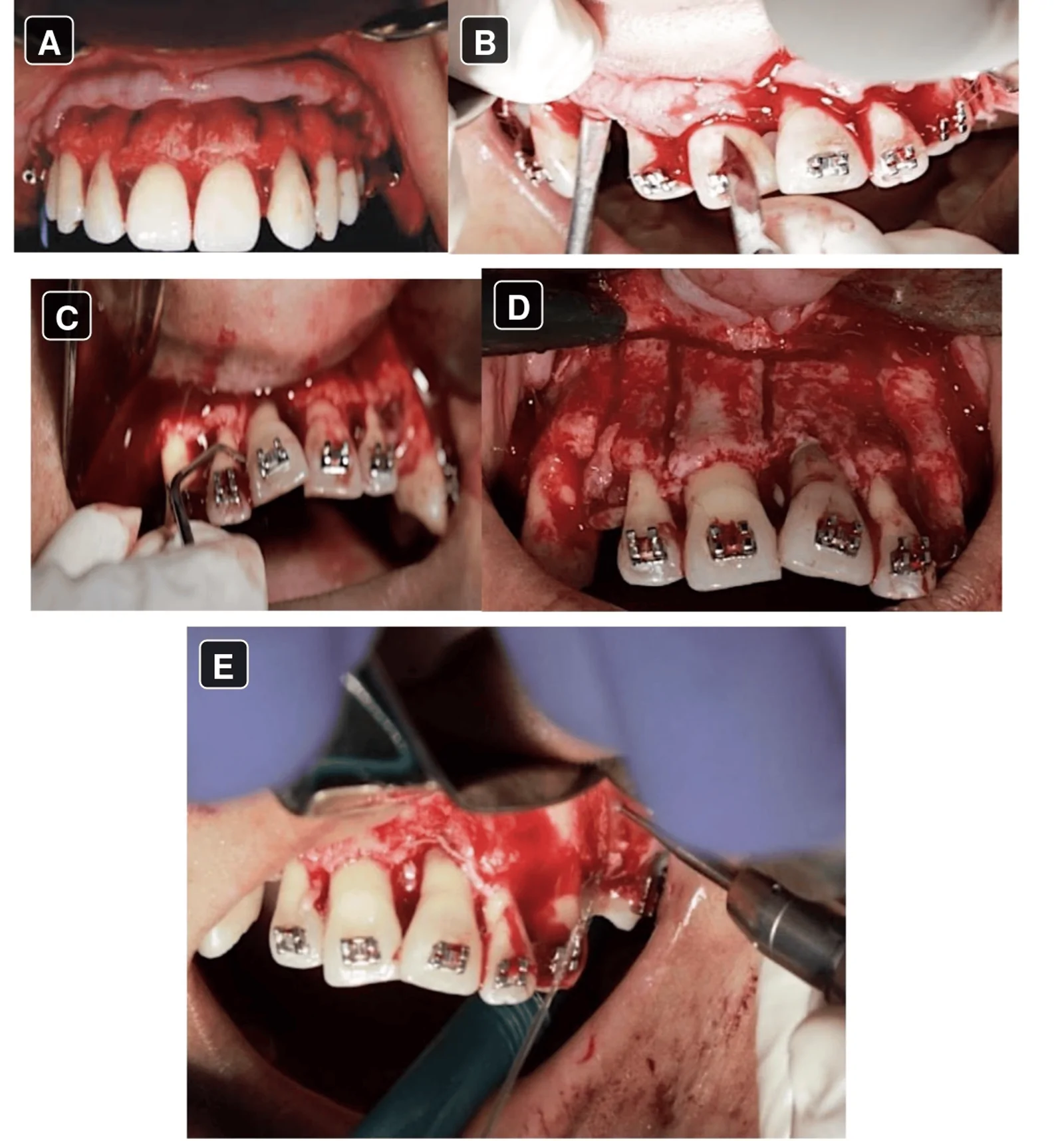
Selective corticotomies in the maxillary anterior region Intraoperative views demonstrate (A-D) vertical interradicular corticotomy cuts in the maxillary anterior region and (E) completion of the corticotomy pattern extending apically beyond the root apices.

Following decortication, 3 cubic centimeters of particulate bone graft material (GenMix®; Bionext Produtos Biotecnológicos Ltda., Mogi Mirim, São Paulo, Brazil) was prepared as shown in Figure 6 and then placed over the corticotomy sites and deficient facial ridge.

**Figure 6:**
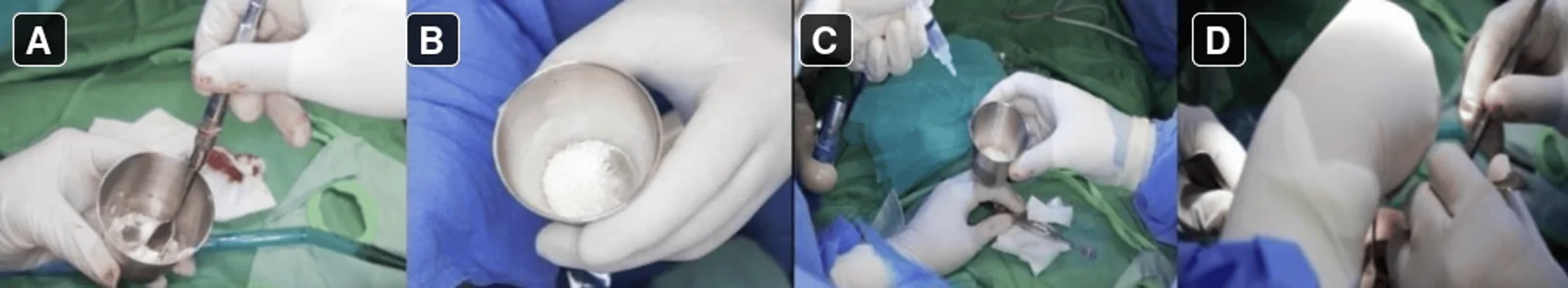
Preparation of the particulate bone graft (A) Addition of the liquid component to the mixing container; (B) Particulate bone graft material before hydration; (C) Hydration of the graft material; (D) Mixing of the components to obtain a homogeneous graft consistency.

A 50 × 37 mm titanium mesh was subsequently adapted and stabilized over the grafted area, as shown in Figure 7. The mesh was used to maintain regenerative space and preserve the intended three-dimensional ridge contour during healing.

**Figure 7:**
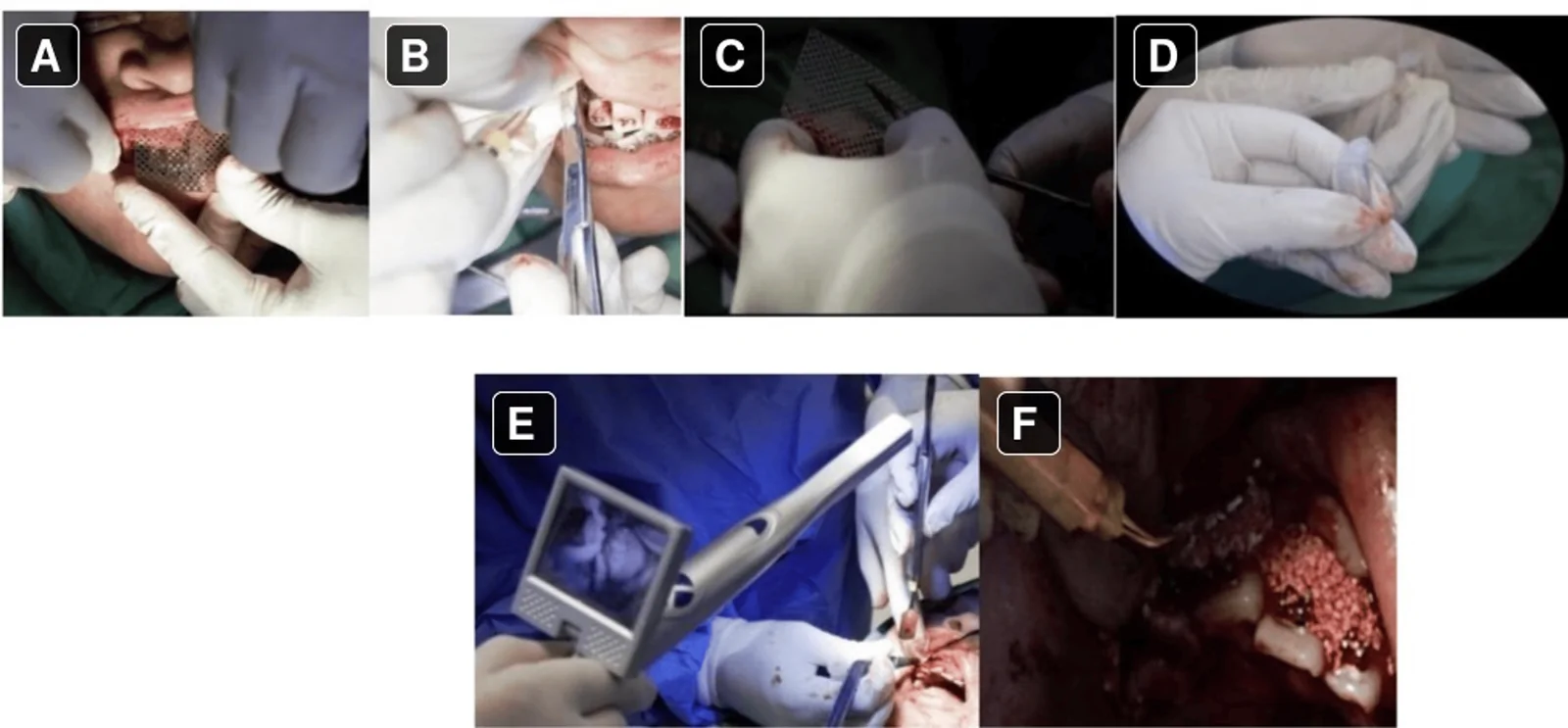
Titanium mesh adaptation (A-C) Intraoperative trimming and contouring of the titanium mesh to conform to the recipient site; (D) Handling of the mesh during preparation; (E) Intraoperative visualization of the surgical field; (F) Particulate bone graft positioned within the recipient area before mesh coverage. The titanium mesh was adapted and stabilized over the grafted area to maintain regenerative space and protect the bone graft during healing.

The flap was advanced and repositioned to permit tension-free primary closure and complete coverage of the regenerative materials. Interrupted sutures were placed to stabilize the surgical site, and healing progressed satisfactorily without recorded intraoperative complications. Flap repositioning and primary closure are shown in Figure 8.

**Figure 8:**
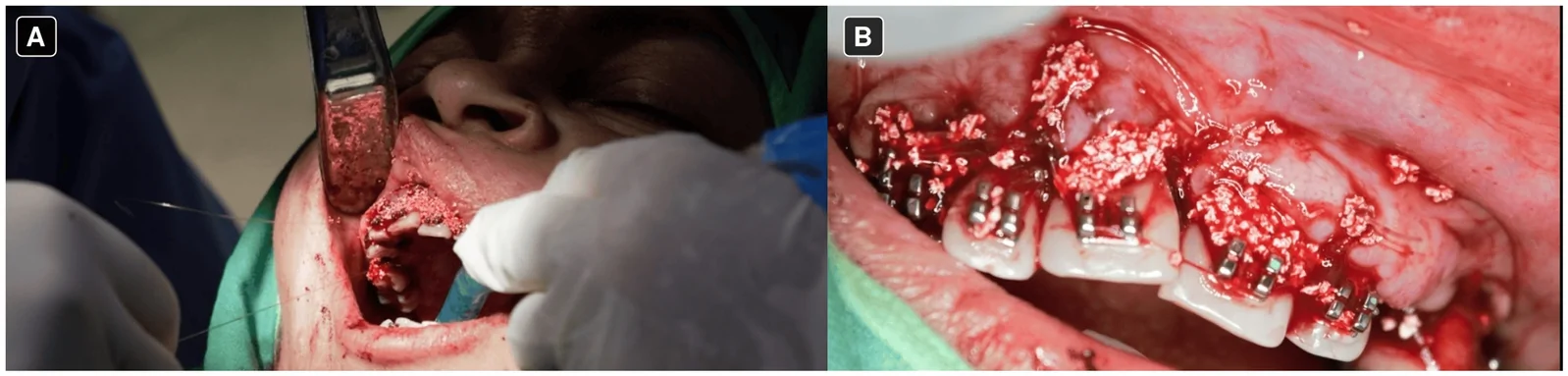
Flap repositioning and primary closure (A) Regenerative site before flap repositioning; (B) Repositioning and approximation of the mucoperiosteal flap with interrupted sutures during primary closure. The flap was advanced without tension to cover the regenerative site.

Postoperative management 

Postoperative care included antibiotic and anti-inflammatory therapy selected according to the treating clinician’s protocol and the patient’s documented allergy profile. The patient received detailed instructions to maintain a liquid diet during the initial healing period and then progress gradually to a soft diet. She was instructed to avoid trauma to the surgical area, smoking, vigorous rinsing, and strenuous physical activity during early healing, and oral-hygiene measures were modified to protect the surgical site while maintaining plaque control elsewhere. 

At the one-day postoperative evaluation, expected facial edema was present without clinically evident extraoral signs of complications. The anonymized postoperative appearance is shown in Figure 9. 

**Figure 9:**
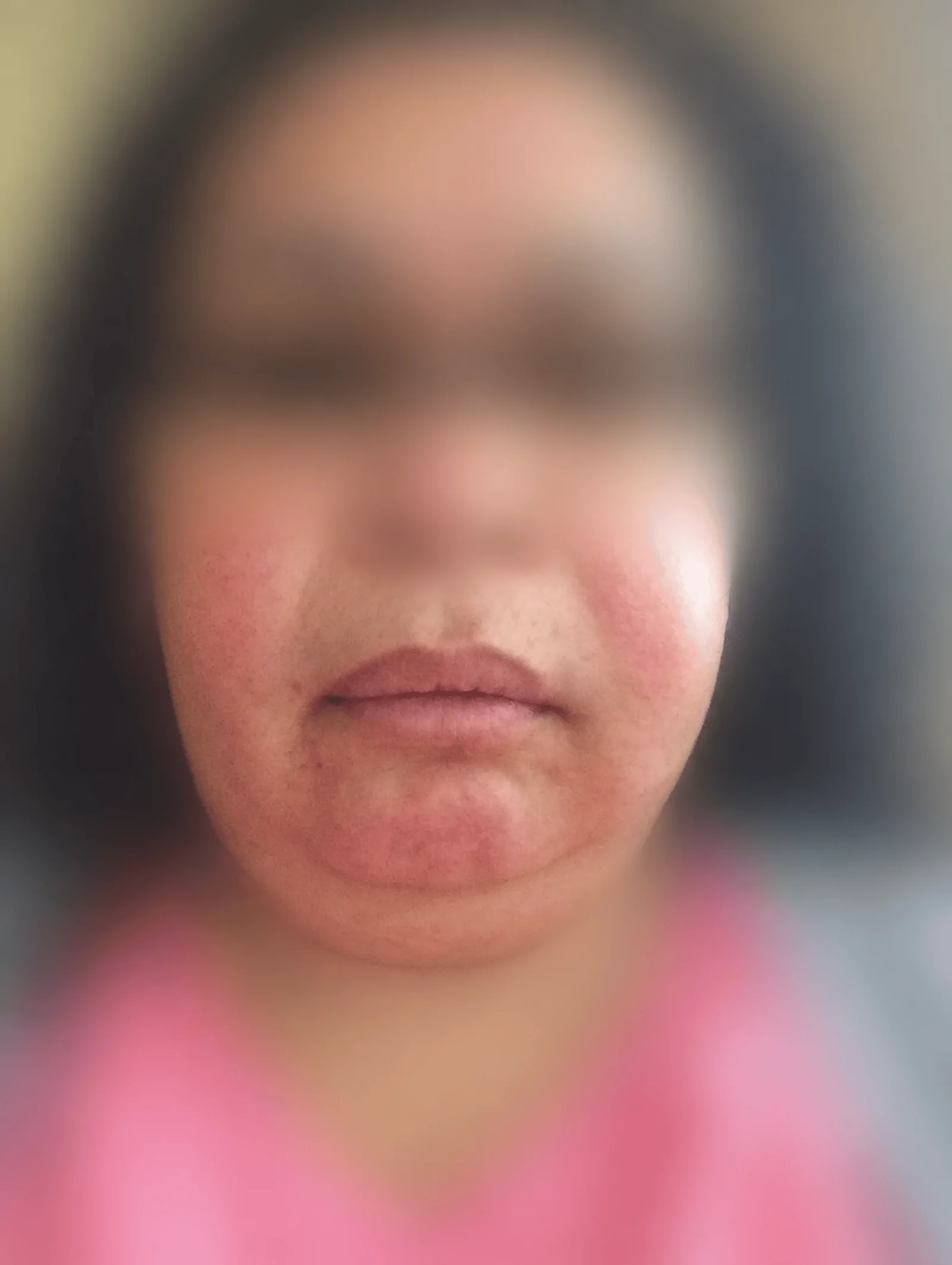
One-day postoperative extraoral appearance An anonymized extraoral frontal view one day after surgery shows expected postoperative facial edema without clinically evident extraoral signs of complications. The patient provided written and signed consent, allowing publication of this identifiable facial image in an open-access journal.

At the eight-day evaluation, the surgical site demonstrated favorable early soft-tissue healing, reduced inflammation, and adequate wound stability without visible dehiscence, as shown in Figure 10. No early postoperative complication was clinically evident at this assessment. 

**Figure 10:**
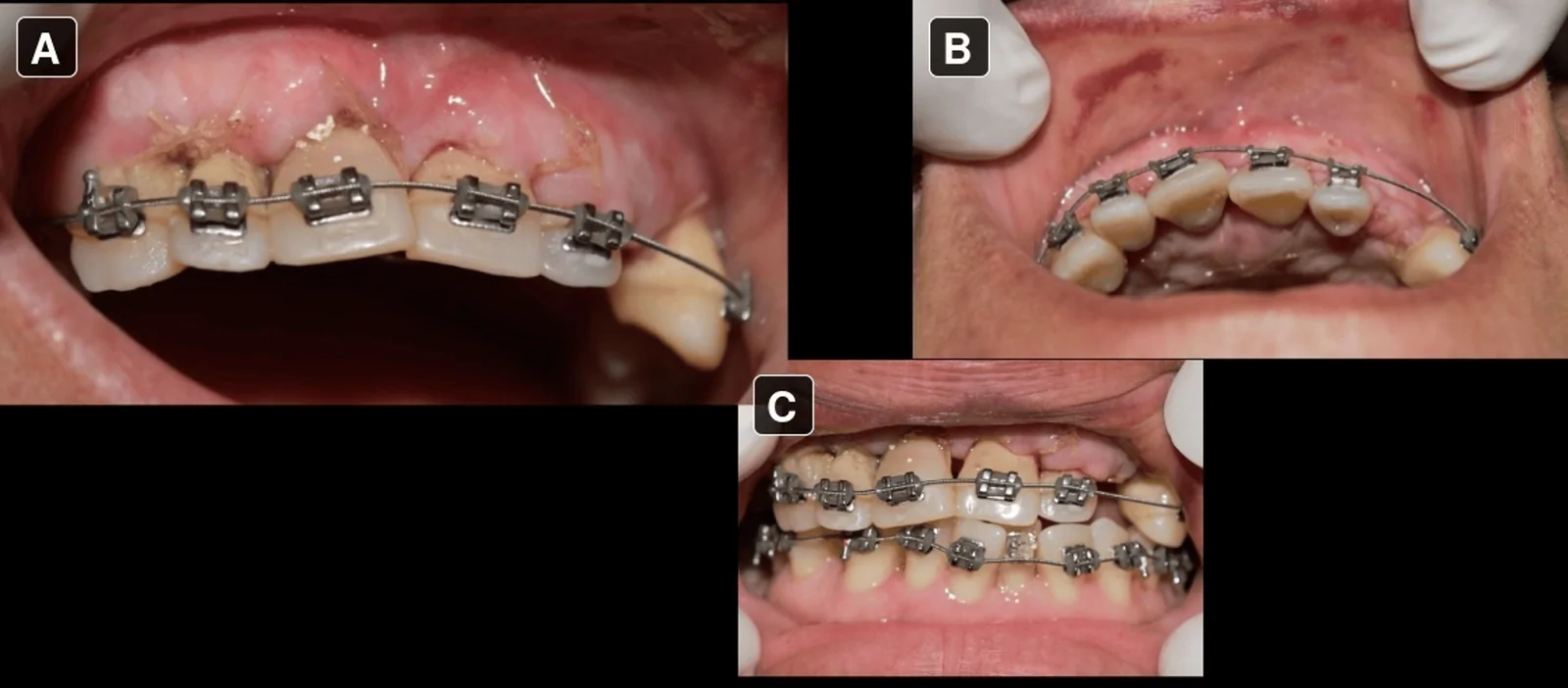
Eight-day postoperative healing (A) Close anterior view of the surgical area; (B) Maxillary occlusal view; (C) Frontal intraoral view. The images demonstrate early soft-tissue healing, reduced postoperative inflammation, and maintenance of wound stability without visible dehiscence.

Clinical examinations during healing assessed wound stability, soft-tissue maturation, periodontal health, and maintenance of the augmented ridge contour. Orthodontic treatment proceeded after periodontal stability and satisfactory early healing of the regenerative site had been established. The titanium mesh remained in place during this phase, and the three-month clinical findings are shown in Figure 11.

**Figure 11:**
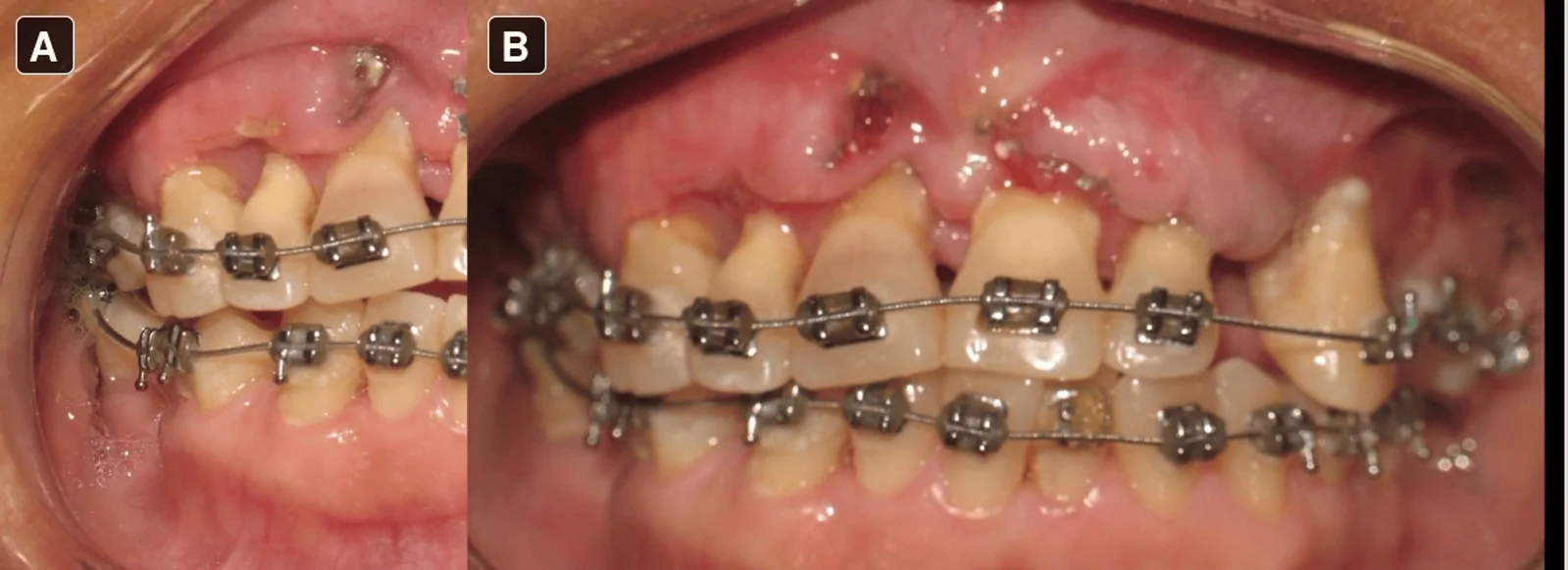
Three-month follow-up (A) Lateral intraoral view; (B) Frontal intraoral view. The images demonstrate satisfactory soft-tissue maturation and maintenance of the augmented alveolar ridge contour during ongoing orthodontic treatment.

At nine months, the titanium mesh was removed after satisfactory clinical healing. The surgical view at removal showed maintenance of the augmented anterior maxillary ridge contour, as shown in Figure 12. Histologic confirmation and standardized three-dimensional measurements of bone regeneration were not obtained. 

**Figure 12:**
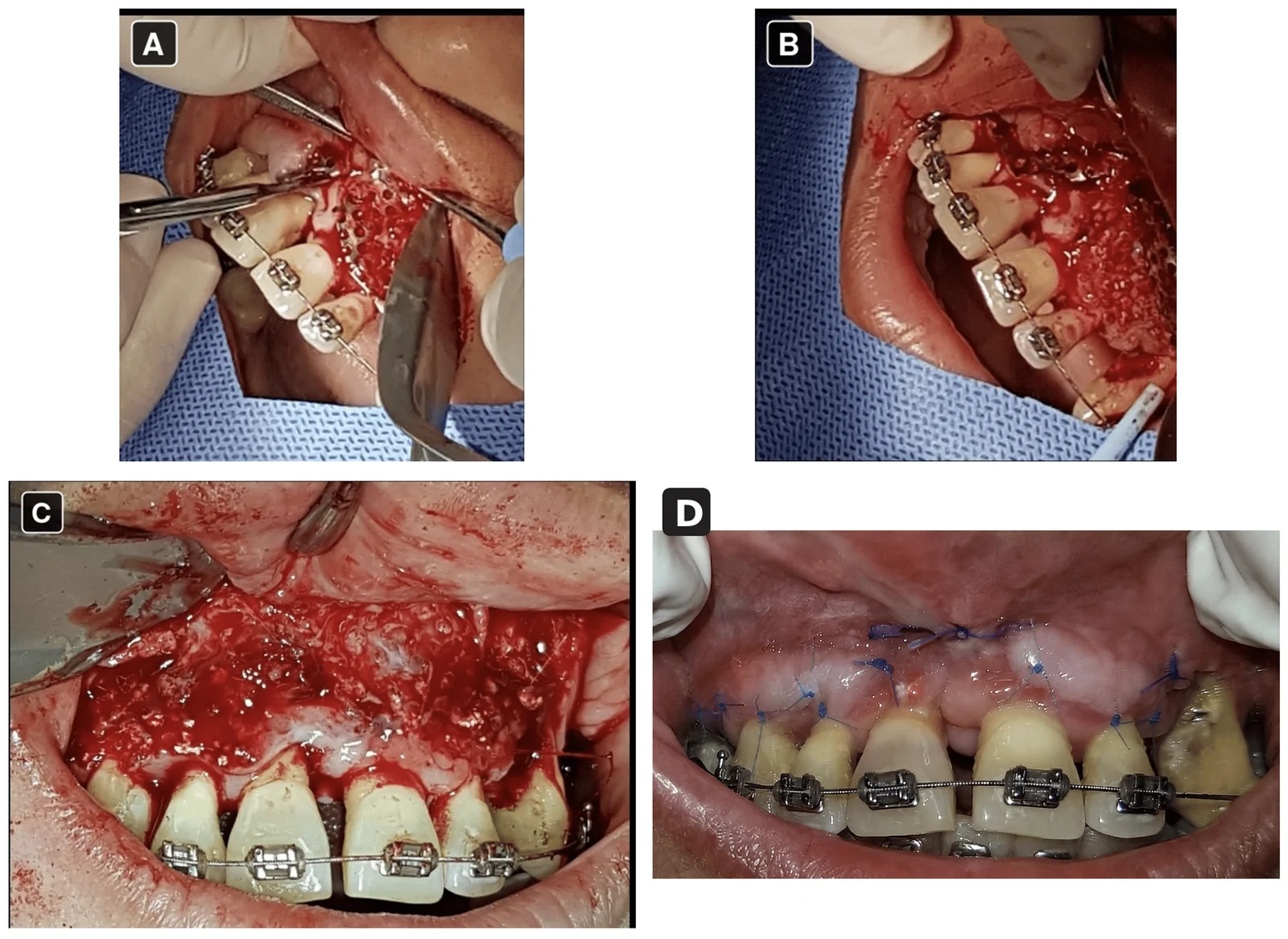
Titanium mesh removal (A) Exposure of the titanium mesh following flap elevation; (B) Removal of the mesh from the treated area; (C) Clinical appearance of the anterior maxillary ridge after mesh removal, showing maintenance of the clinically apparent augmented ridge contour; (D) Flap repositioning and primary closure with sutures following mesh removal. Histologic confirmation and standardized three-dimensional measurements were not obtained.

Clinical outcome 

During the documented follow-up, no recurrent acute inflammation, additional tooth loss, mesh exposure, wound dehiscence, or other clinically evident postoperative complication was recorded. Because complete serial periodontal measurements were unavailable, absence of progressive attachment loss could not be quantified. Clinical views obtained during orthodontic treatment are shown in Figure 13.

**Figure 13:**
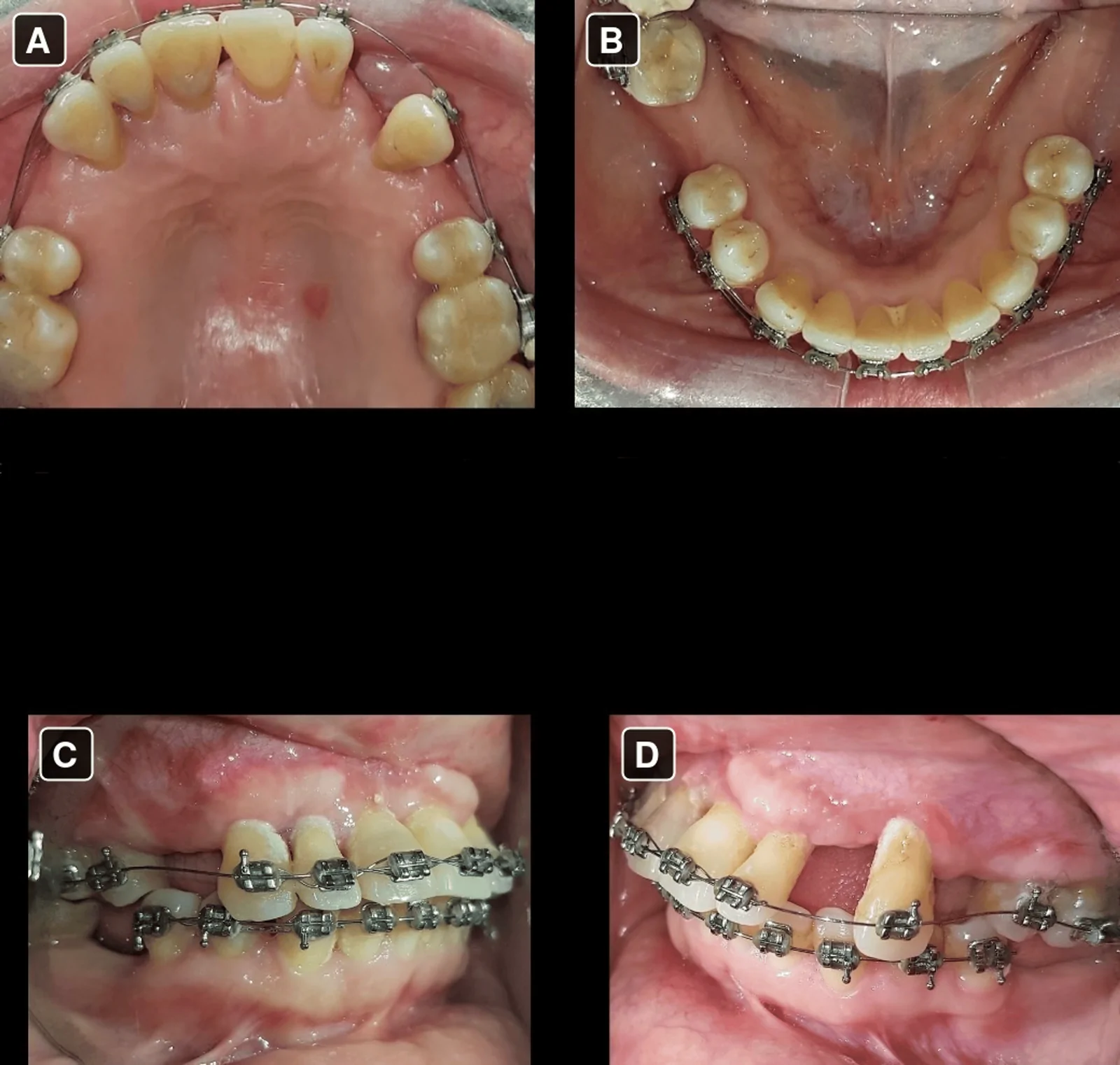
Orthodontic treatment progress (A) Maxillary occlusal view; (B) Mandibular occlusal view; (C) Right lateral view; (D) Left lateral view. The images demonstrate ongoing dental alignment and controlled movement of the anterior teeth while maintaining the augmented maxillary ridge.

Subsequent clinical photographs showed continued orthodontic alignment and maintenance of the clinically apparent augmented anterior maxillary ridge contour. Frontal and occlusal views documenting this clinical course are shown in Figure 14.

**Figure 14:**
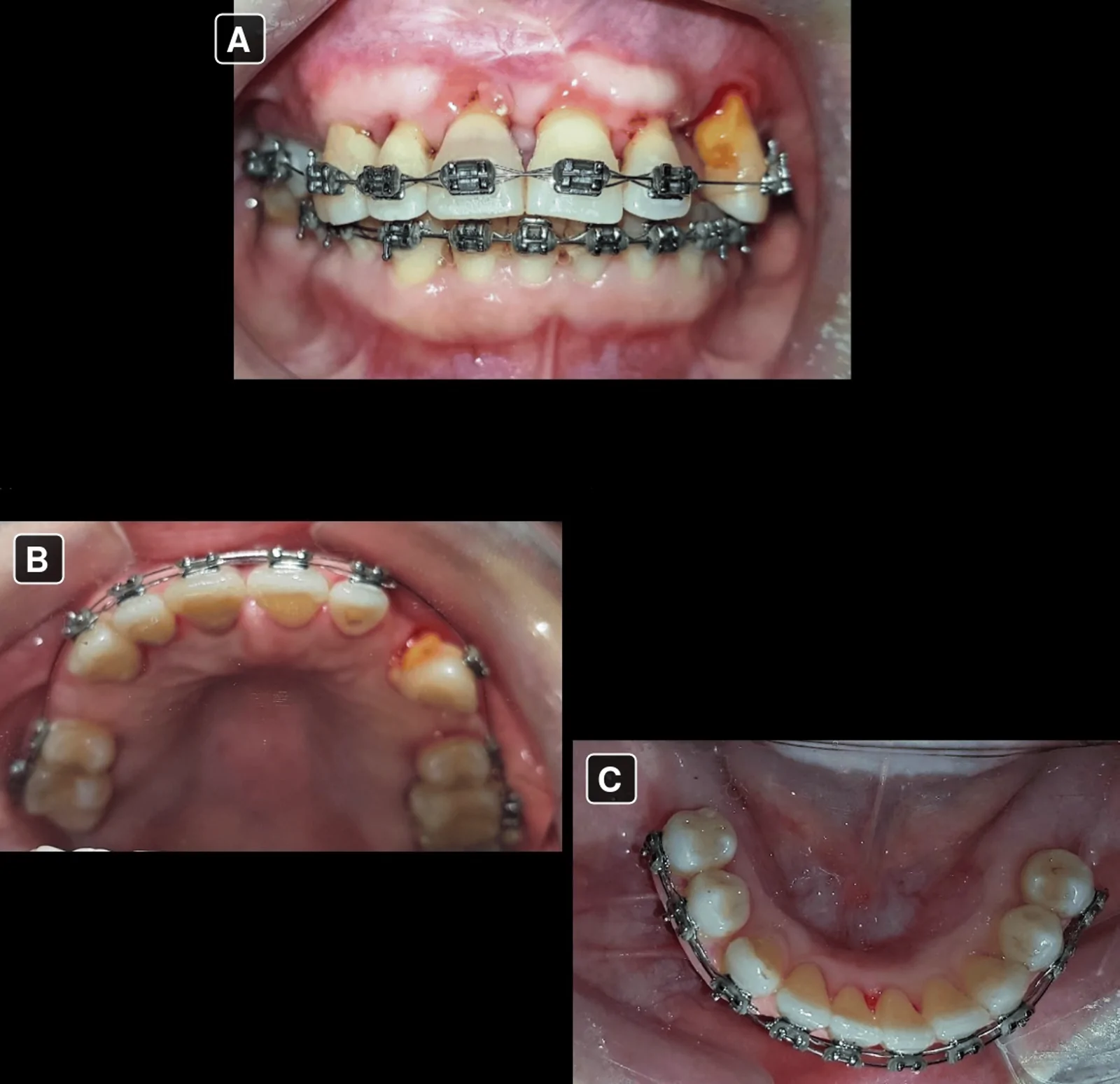
Clinical and orthodontic follow-up (A) Frontal view demonstrating continued orthodontic alignment and periodontal stability; (B) Maxillary occlusal view showing the maintained anterior maxillary ridge; (C) Mandibular occlusal view demonstrating ongoing orthodontic treatment.

At the four-month orthodontic follow-up, continued tooth movement and progressive improvement in maxillary anterior alignment were observed. Frontal, occlusal, and lateral intraoral views documented ongoing space redistribution and no clinically apparent loss of the augmented ridge contour, as shown in Figure 15. 

**Figure 15:**
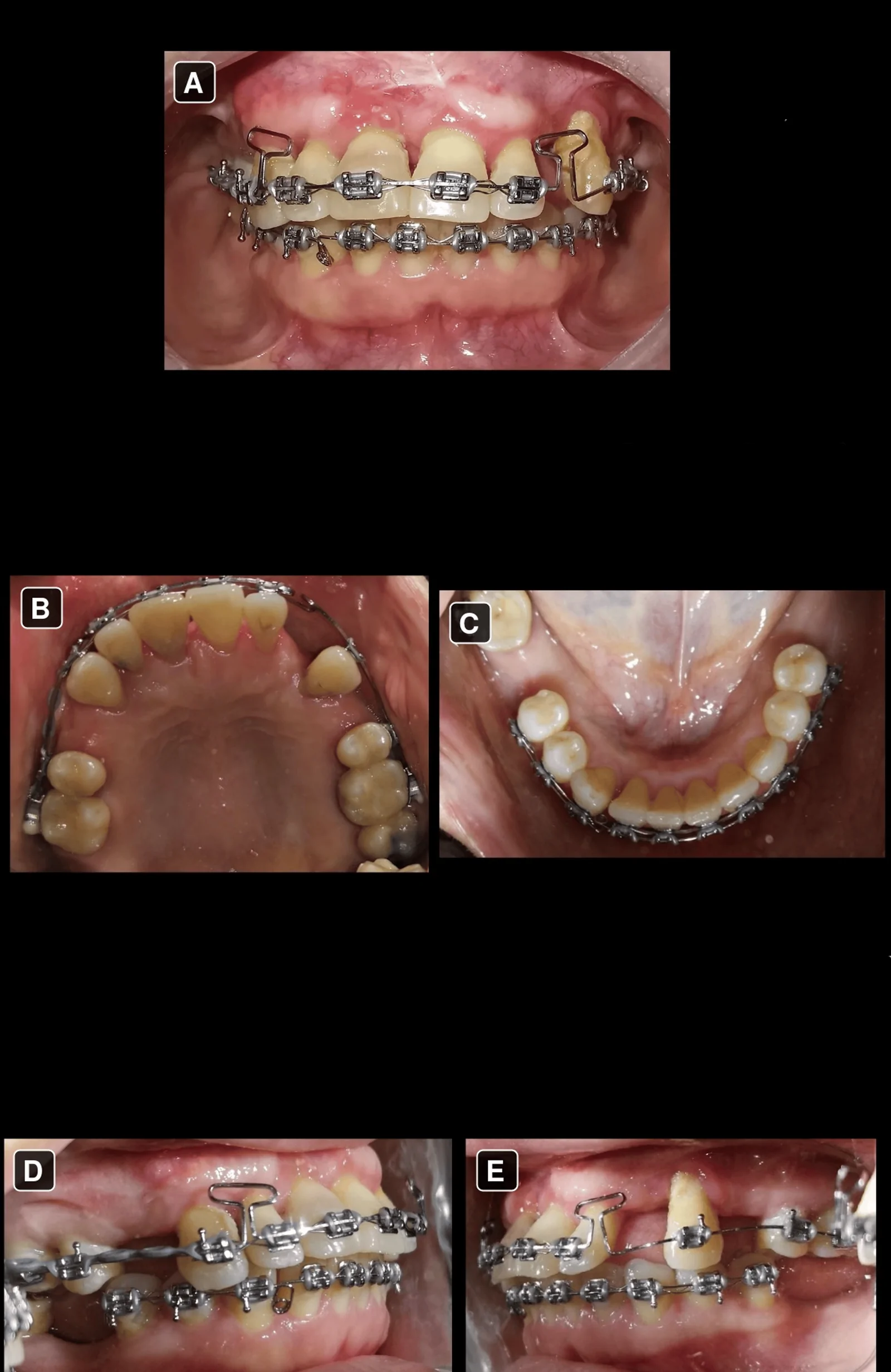
Four-month orthodontic follow-up (A) Frontal view showing continued alignment of the maxillary anterior teeth; (B) Maxillary occlusal view; (C) Mandibular occlusal view; (D) Right lateral view; (E) Left lateral view. The images demonstrate progressive orthodontic tooth movement and maintenance of periodontal stability during treatment.

A panoramic radiograph and periapical radiographs were obtained during the same four-month follow-up to evaluate tooth positioning and the radiographic appearance of the treated anterior maxillary region. These radiographic findings are shown in Figure 16. Because standardized preoperative and postoperative cone-beam computed tomography or other three-dimensional measurements were unavailable, neither bone gain nor the composition of the augmented tissue could be quantified. The regenerative outcome was therefore assessed descriptively from serial clinical photographs and conventional radiographs. These records documented maintenance of the clinically apparent augmented ridge contour and the radiographic appearance of the treated region, but they do not establish histologic bone regeneration or a measured increase in ridge dimensions.

**Figure 16:**
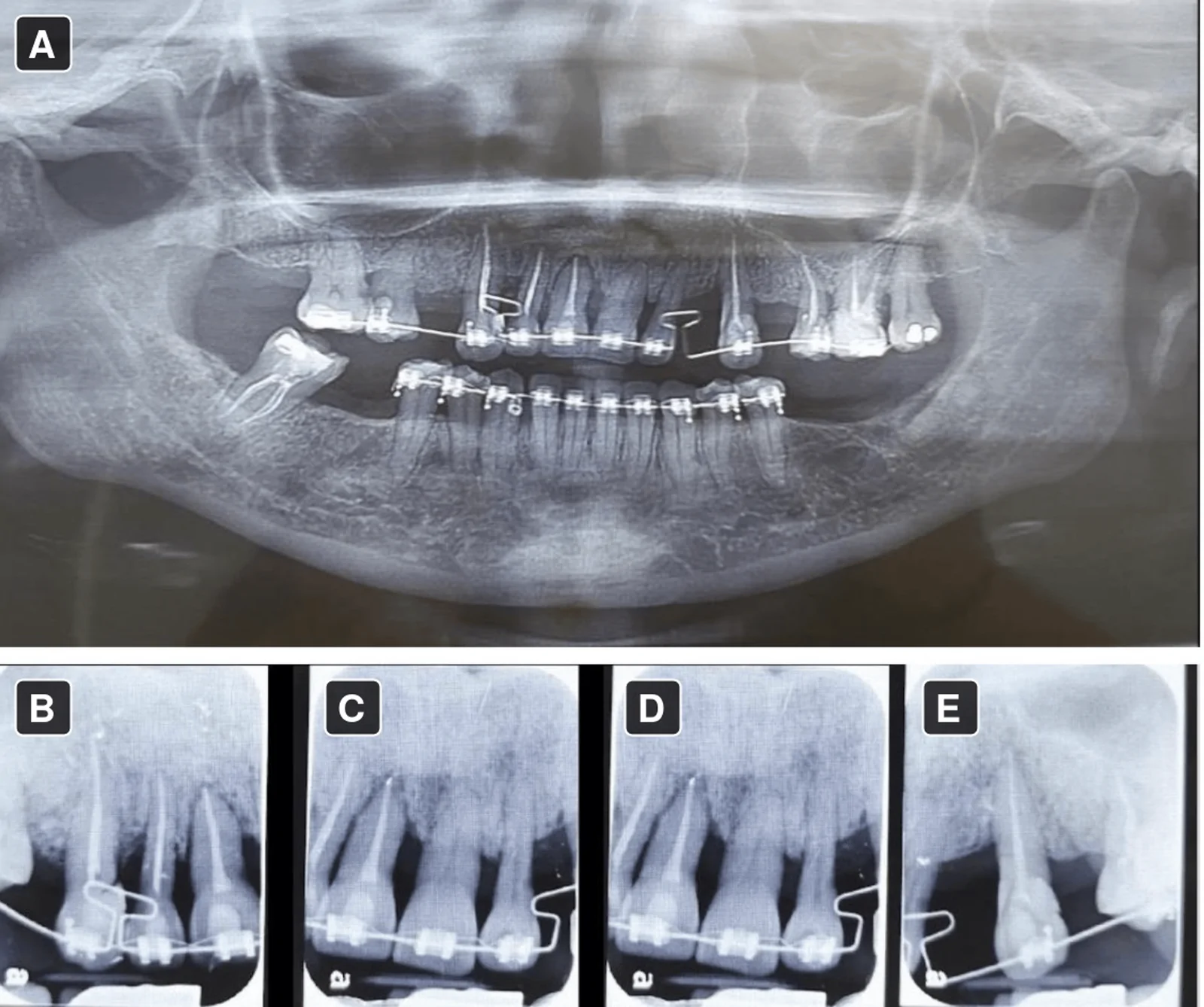
Four-month radiographic follow-up (A) Panoramic radiograph showing overall dental status and orthodontic tooth positioning; (B-E) Periapical radiographs of the maxillary anterior region showing the radiographic appearance of the treated area during ongoing orthodontic treatment. These nonstandardized two-dimensional images were used for descriptive follow-up and do not quantify bone gain.

Implant-supported rehabilitation was recommended as a future restorative phase, subject to definitive three-dimensional assessment and restorative evaluation. This report focuses on the interdisciplinary management undertaken to improve the biological and anatomical conditions before that phase. 

Rationale for the selected treatment approach 

The interdisciplinary strategy was selected to address the patient’s biological, functional, and esthetic needs while preserving strategically maintainable teeth whenever possible. At the initial examination, generalized periodontal inflammation, advanced attachment loss, severe anterior maxillary alveolar ridge deficiency, pathological tooth migration, and occlusal instability precluded immediate orthodontic treatment. 

Extraction of the remaining compromised teeth followed by extensive implant-supported rehabilitation was considered. However, this approach could have resulted in further collapse of the residual alveolar ridge, increased surgical and restorative complexity, and unnecessary removal of teeth considered maintainable after periodontal stabilization. A tooth-preserving strategy involving sequential periodontal therapy, regenerative surgery, controlled orthodontic correction, and supportive periodontal maintenance was therefore selected. 

Initial periodontal therapy was necessary to control active inflammation before surgical or orthodontic intervention. After periodontal stability was achieved, PAOO was combined with GBR to stimulate regional bone remodeling while simultaneously increasing the volume of the deficient ridge. Titanium mesh was selected to provide rigid space maintenance and stabilize the particulate graft during healing, and orthodontic treatment proceeded only after inflammation had been controlled and satisfactory early soft-tissue healing had been confirmed. 

Supportive periodontal therapy at three-month intervals was maintained throughout orthodontic treatment. These visits reinforced plaque control, permitted monitoring of periodontal stability, and supported clinical evaluation of the regenerated tissues. 

## Discussion

The management of this patient illustrates the potential value of a carefully sequenced interdisciplinary approach for advanced periodontitis associated with severe anterior maxillary alveolar ridge deficiency. Instead of immediately extracting all compromised teeth, treatment focused on controlling periodontal inflammation, preserving strategically maintainable teeth, and establishing more favorable conditions for controlled orthodontic tooth movement. 

PAOO combines selective alveolar corticotomies with particulate bone grafting. The surgical injury produced by corticotomy is associated with the RAP, a temporary increase in local bone turnover and remodeling that may facilitate orthodontic tooth movement [8]. When appropriately indicated, the addition of bone grafting may increase alveolar bone volume and improve the anatomical housing available for controlled orthodontic tooth movement; however, outcomes depend on periodontal stability, surgical technique, orthodontic biomechanics, and patient-related factors. 

In this patient, GBR using a particulate bone graft and titanium mesh was associated with maintenance of an augmented anterior maxillary ridge contour. Titanium mesh provides rigid space maintenance, stabilizes the particulate graft, and supports preservation of the intended three-dimensional contour; however, the amount and composition of newly formed bone were not measured. Its use is technique-sensitive, and complications such as soft-tissue dehiscence and mesh exposure have been reported [12-14]. Careful flap design, adequate soft-tissue release, stable fixation, and tension-free primary closure are therefore important components of treatment. 

The biological response to alveolar grafting depends on coordinated inflammatory, vascular, cellular, and remodeling events. Macrophage polarization and other osteoimmune signals can influence angiogenesis, osteogenic differentiation, and the balance between bone formation and resorption, while scaffold architecture and surface properties affect cellular attachment and tissue integration [18,19]. These principles are particularly relevant in a periodontally compromised site, where residual dysbiosis or uncontrolled inflammation may impair healing. In this case, initial periodontal therapy and continuing plaque control were therefore treated as prerequisites for surgery and orthodontic movement. Nevertheless, no inflammatory biomarkers, immune-cell phenotypes, histologic specimens, or material-specific immunomodulatory effects were evaluated; any biological interpretation beyond the observed clinical course remains inferential. 

Another important aspect of this case was the decision to delay orthodontic treatment until periodontal inflammation had been controlled and satisfactory healing of the regenerative site had been achieved. Orthodontic tooth movement in the presence of active periodontal inflammation may contribute to additional attachment loss [15]. In contrast, carefully controlled orthodontic treatment may be performed in periodontally compromised patients when inflammation is controlled, appropriate biomechanics are used, and periodontal maintenance is maintained [15]. 

The favorable clinical course was associated with integration of periodontal therapy, regenerative surgery, orthodontics, and continuing supportive care rather than with a single procedure. Supportive periodontal therapy at three-month intervals reinforced oral hygiene and permitted periodontal monitoring during active orthodontic treatment [16,17]. 

Although the observed clinical course was favorable, this single case should be interpreted cautiously. The available records support maintenance of the clinically apparent augmented ridge contour, progressive orthodontic alignment, and the absence of recorded acute complications during the documented follow-up. They do not objectively quantify bone gain, demonstrate histologic regeneration, or establish predictable treatment effectiveness. Prospective studies using complete serial periodontal measurements, standardized cone-beam computed tomography or other three-dimensional analyses, patient-reported outcomes, and larger patient populations are needed to evaluate the effectiveness and predictability of this approach.

Clinical lessons 

Active periodontal inflammation should be controlled before orthodontic treatment is initiated in patients with advanced periodontitis. In carefully selected patients, PAOO combined with GBR may improve the biological and anatomical conditions for controlled orthodontic movement. Titanium mesh can provide effective space maintenance when substantial horizontal and vertical ridge augmentation is required; however, its use is technique-sensitive and requires careful soft-tissue management. 

Orthodontic forces should be carefully controlled and accompanied by close periodontal monitoring. Supportive periodontal therapy and patient adherence are essential for maintaining stability throughout interdisciplinary treatment. Successful management also requires coordinated treatment planning among periodontists, orthodontists, restorative dentists, and the patient. 

Follow-up 

The patient remained enrolled in supportive periodontal therapy at regular intervals throughout active orthodontic treatment. During the available follow-up, no recurrent acute inflammation or additional tooth loss was documented; however, complete serial measurements were unavailable to quantify changes in attachment level. Clinical photographs and conventional radiographs documented maintenance of the augmented anterior maxillary ridge contour and progressive orthodontic alignment during the available follow-up.

Future implant-supported rehabilitation remained planned, subject to definitive three-dimensional assessment and restorative evaluation. The available records did not document completion of this restorative phase. 

Limitations 

This report describes one patient; therefore, its findings cannot be generalized to all patients with advanced periodontitis and severe alveolar ridge deficiency. Standardized preoperative and postoperative cone-beam computed tomography or other three-dimensional measurements of ridge width and height were unavailable, preventing objective quantification of bone gain. Histologic evaluation was not performed; consequently, the composition of the augmented tissue could not be determined.

Complete numerical periodontal parameters were not available at every follow-up visit, including final full-mouth probing depths, clinical attachment levels, bleeding on probing, mobility grades, and plaque scores. Exact dates for all treatment phases and the interval between surgery and orthodontic force activation were also unavailable. Outcome assessment was therefore based primarily on the available clinical records, serial photographs, periodontal charts, and conventional radiographs. Implant-supported rehabilitation was recommended as a future restorative phase, but its completion was not documented. 

## Conclusions

This case documents a carefully sequenced interdisciplinary approach integrating periodontal stabilization, selective alveolar corticotomy, titanium mesh-supported GBR, controlled orthodontic treatment, and supportive periodontal therapy. During the available follow-up, the records showed preservation of strategically maintainable teeth, progressive orthodontic alignment, maintenance of the clinically apparent augmented anterior maxillary ridge contour, and no recorded acute postoperative complications.

These observations are limited to one patient and do not quantify bone gain, confirm histologic regeneration, or establish the efficacy or predictability of the treatment approach. The clinical sequence may be considered in carefully selected and adherent patients after individualized periodontal, surgical, orthodontic, and restorative assessment, with close periodontal maintenance and objective three-dimensional evaluation whenever available.

## References

[1] Caton JG, Armitage G, Berglundh T (2018). A new classification scheme for periodontal and peri-implant diseases and conditions - introduction and key changes from the 1999 classification. J Periodontol.

[2] Tonetti MS, Greenwell H, Kornman KS (2018). Staging and grading of periodontitis: framework and proposal of a new classification and case definition. J Periodontol.

[3] Papapanou PN, Sanz M, Buduneli N (2018). Periodontitis: Consensus report of Workgroup 2 of the 2017 World Workshop on the Classification of Periodontal and Peri-Implant Diseases and Conditions. J Clin Periodontol.

[4] Lang NP, Bartold PM (2018). Periodontal health. J Clin Periodontol.

[5] Wilcko WM, Wilcko T, Bouquot JE, Ferguson DJ (2001). Rapid orthodontics with alveolar reshaping: two case reports of decrowding. Int J Periodontics Restorative Dent.

[6] Murphy KG, Wilcko MT, Wilcko WM, Ferguson DJ (2009). Periodontal accelerated osteogenic orthodontics: a description of the surgical technique. J Oral Maxillofac Surg.

[7] Wilcko MT, Wilcko WM, Pulver JJ, Bissada NF, Bouquot JE (2009). Accelerated osteogenic orthodontics technique: a 1-stage surgically facilitated rapid orthodontic technique with alveolar augmentation. J Oral Maxillofac Surg.

[8] Frost HM (1983). The regional acceleratory phenomenon: a review. Henry Ford Hosp Med J.

[9] Sculean A, Nikolidakis D, Schwarz F (2008). Regeneration of periodontal tissues: combinations of barrier membranes and grafting materials - biological foundation and preclinical evidence: a systematic review. J Clin Periodontol.

[10] Lang NP, Lindhe J (2015). Clinical Periodontology and Implant Dentistry. https://www.wiley.com/en-us/Clinical%2BPeriodontology%2Band%2BImplant%2BDentistry%2C%2B2%2BVolume%2BSet%2C%2B6th%2BEdition-p-9781118940471.

[11] Urban IA, Monje A, Lozada J, Wang HL (2017). Principles for vertical ridge augmentation in the atrophic posterior mandible: a technical review. Int J Periodontics Restorative Dent.

[12] Rakhmatia YD, Ayukawa Y, Furuhashi A, Koyano K (2013). Current barrier membranes: titanium mesh and other membranes for guided bone regeneration in dental applications. J Prosthodont Res.

[13] Aceves-Argemí R, Roca-Millan E, González-Navarro B, Marí-Roig A, Velasco-Ortega E, López-López J (2021). Titanium meshes in guided bone regeneration: a systematic review. Coatings.

[14] Li S, Zhao J, Xie Y, Tian T, Zhang T, Cai X (2021). Hard tissue stability after guided bone regeneration: a comparison between digital titanium mesh and resorbable membrane. Int J Oral Sci.

[15] Ong MM, Wang HL (2002). Periodontic and orthodontic treatment in adults. Am J Orthod Dentofacial Orthop.

[16] Lang NP, Tonetti MS (2003). Periodontal risk assessment (PRA) for patients in supportive periodontal therapy (SPT). Oral Health Prev Dent.

[17] Axelsson P, Lindhe J (1981). The significance of maintenance care in the treatment of periodontal disease. J Clin Periodontol.

[18] Huang X, Lou Y, Duan Y, Liu H, Tian J, Shen Y, Wei X (2024). Biomaterial scaffolds in maxillofacial bone tissue engineering: a review of recent advances. Bioact Mater.

[19] Tang M, Zhang L, Hieawy A, Liang S, Liu H, Shen Y (2026). Regulatory network-oriented design of stimuli-responsive biomaterials for oral and craniofacial bone regeneration. Mater Des.

